# Family Routines and Parenting Stress in Families of Children With and Without Intellectual Disabilities: A Cross-Sectional Study

**DOI:** 10.3390/healthcare14193153

**Published:** 2026-09-23

**Authors:** Vesela Milankov, Jelena Radonjić, Tatjana Krstić, Jovana Uzelac, Sandra Glamočak, Ivana Matić, Špela Golubović

**Affiliations:** 1 Department of Special Education and Rehabilitation, Faculty of Medicine, University of Novi Sad, 21000 Novi Sad, Serbia; jelena.radonjic@mf.uns.ac.rs (J.R.); jovana.uzelac@mf.uns.ac.rs (J.U.); sandra.glamocak@mf.uns.ac.rs (S.G.); ivana.matic@mf.uns.ac.rs (I.M.); spela.golubovic@mf.uns.ac.rs (Š.G.); 2 Department of Psychology, Faculty of Medicine, University of Novi Sad, 21000 Novi Sad, Serbia; tatjana.krstic@mf.uns.ac.rs

**Keywords:** parenting stress, family routines, family resilience, parental well-being

## Abstract

**Highlights:**

**What are the main findings?**

- Parents of children with intellectual disabilities reported substantially higher parenting stress and less frequent family routines across all assessed domains compared with parents of typically developing children.
- In both groups, more frequent family routines were associated with lower parenting stress, while pooled analyses showed that routine frequency remained independently associated with parenting stress after accounting for study group and selected sociodemographic and family characteristics.

**What are the implications of the main findings?**

- Routine-based, family-centered approaches that support everyday family organization may represent a potentially useful avenue for supporting parental well-being, particularly in families of children with intellectual disabilities.
- Clinicians and service providers should systematically assess and support family routines, tailoring recommendations to the child’s developmental abilities and the family’s contextual resources to promote adaptation and resilience.

**Abstract:**

**Background:** Families of children with intellectual disabilities often experience increased caregiving demands that may contribute to elevated parenting stress. Family routines represent an important aspect of everyday family organization and may support adaptation by providing structure, predictability, and continuity. However, the role of family routines in relation to parenting stress among families of children with intellectual disabilities remains insufficiently understood. This study aimed to examine parenting stress, the frequency and perceived importance of family routines in families of children with and without intellectual disabilities, and the associations between family routines and parenting stress. **Methods:** A cross-sectional study was conducted among parents of children with intellectual disabilities and parents of typically developing children. Family routines were assessed using the Family Time and Routines Index (FTRI), and parenting stress was assessed using the Parental Stress Scale (PSS). Group differences were examined using appropriate nonparametric and categorical-data tests, while associations between parenting stress and family routines were examined using Spearman rank-order correlations separately by study group. A hierarchical multiple regression analysis was conducted in the pooled sample to examine the incremental contribution of family routines to parenting stress beyond study group and selected sociodemographic and family characteristics. Group routine interaction terms were additionally tested to examine whether these associations differed between the TD and ID groups. **Results:** Parents of children with intellectual disabilities reported significantly higher levels of parenting stress compared with parents of typically developing children. Significant differences were observed in the frequency of family routines, with parents of typically developing children reporting more frequent routines across assessed domains. Higher frequency of family routines was significantly associated with lower levels of parenting stress in both groups. In the pooled regression analysis, family routine frequency and perceived importance together explained an additional 6.6% of the variance in parenting stress beyond study group and selected sociodemographic and family characteristics. In the final model, greater family routine frequency was independently associated with lower parenting stress, whereas perceived importance did not make a significant independent contribution. No significant group × routine interactions were observed. **Conclusions:** The findings suggest that the regular organization of family routines represents an important aspect of family functioning associated with parental well-being. Supporting families in establishing and maintaining adaptive routines may represent a valuable component of family-centered interventions for reducing parenting stress and strengthening everyday family functioning, particularly among families of children with intellectual disabilities. Longitudinal and intervention studies are needed to further clarify the role of family routines in supporting family adaptation.

## 1. Introduction

The family constitutes a child’s primary developmental environment and is an important context for child development. From a family systems perspective, everyday family functioning reflects not only the characteristics of individual family members but also the ways in which families organize daily life and adapt to developmental transitions and life changes [1]. Within this framework, family routines can be understood as predictable and recurring patterns of activities and interactions that structure everyday family life and contribute to a sense of stability and continuity [2,3]. Family routines are not merely a means of organizing everyday life; they can also serve as a protective resource by providing regularity and predictability. More consistent routines have been associated with lower parenting stress and fewer bedtime-resistance behaviors in young children, while a higher frequency of family routines has been linked to fewer externalizing problems among children with neurodevelopmental delays. During periods of major disruption, such as the COVID-19 pandemic, changes in family routines and family dynamics were also related to parent and child psychological well-being and family resilience processes [4,5,6]. However, family routines are neither static nor equally functional across families. Their frequency and form may vary across developmental stages and cultural contexts and may be altered by family demands, including chronic health conditions and other stressful life circumstances. Accordingly, family routines can be understood as dynamic patterns that families may modify in response to changing circumstances, while continuing to support organization, adaptation, and a sense of normalcy in everyday life [2,3,7].

One of the factors that may substantially influence the maintenance of family routines is parental stress. Contemporary conceptualizations describe parental stress as the result of a complex interaction among parents’ individual characteristics, children’s needs, family relationships, and the broader social environment. Prolonged exposure to parental stress has been associated with poorer parental mental health, lower parenting satisfaction, less consistent parenting practices, and impaired family functioning [8]. Predictable and consistent family routines may contribute to better organization of everyday life, reduce uncertainty, and help parents cope more effectively with daily demands and stress [3,4,8]. Higher levels of parental stress may make family routines more difficult to maintain, particularly under conditions of substantial disruption and increased daily demands. Conversely, regular engagement in structured family routines may serve as a protective resource, being associated with lower parental stress in some families and with better family functioning and resilience during periods of stress [6,9].

Families of children with intellectual disabilities may encounter caregiving demands that extend beyond those commonly experienced in parenting. In particular, parents of children with moderate to profound intellectual disabilities often assume substantial direct caregiving responsibilities, and these demands may be compounded by the child’s medical needs, level of intellectual disability, and challenging behaviors. Such caregiving demands are associated with elevated parental stress and poorer family quality of life, highlighting the need to consider the support needs of the whole family [10,11,12]. These caregiving demands may place substantial emotional and practical demands on families and are associated with higher parental stress, poorer family interactions, and lower parental and family quality of life. Families supporting a child with a disability may also face an elevated risk of financial hardship and persistent or recurrent poverty, although these patterns are shaped by broader family and social resources [10,11,12,13]. Child and diagnosis-related factors, socioeconomic circumstances, social isolation, and limited support from partners, family members, social networks, and professionals have been identified as factors associated with higher parental stress among parents of children with special educational needs [8]. Accordingly, compared with parents of typically developing children, parents of children with intellectual and developmental disabilities experience higher levels of parenting stress. Elevated stress may adversely affect parental health and is associated with poorer emotional well-being and less favorable family interactions. In a study of parents of children with intellectual disabilities in Pakistan, a substantial proportion reported symptoms consistent with anxiety, depression, or both; this finding should be interpreted within its specific cultural and service context [10,14,15].

Maintaining consistent family routines may be challenging for families raising children with developmental disabilities, particularly when children’s needs and characteristics require families to modify everyday activities. Research involving families of children with autism spectrum disorder and caregivers of children with disabilities suggests that child-related developmental and behavioral characteristics, together with broader contextual demands, may influence how family routines are organized, negotiated, and adapted [16,17].

At the same time, contemporary research indicates that families differ in how they respond to these challenges. Family responses are shaped not only by the severity of a child’s difficulties but also by the interplay of individual and contextual factors, including family relationships, social support, coping strategies, and the everyday organization of family life [18].

According to Family Resilience Theory, families differ not only in the stressors they encounter but also in their capacity to mobilize available resources and adapt to adverse circumstances. Within this framework, organizational processes such as flexibility, shared roles and responsibilities, and the maintenance of meaningful daily routines may serve as protective resources that support family stability and psychological well-being [1].

Similarly, the Family Adjustment and Adaptation Response (FAAR) model emphasizes that successful family adaptation depends on the balance between the demands experienced by the family and the capabilities and resources available to manage those demands. When family demands exceed available capabilities and resources, the risk of parental and family stress may increase. From this perspective, family routines may be conceptualized as organizational resources that help structure everyday life and facilitate the effective use of family resources, thereby potentially supporting adaptation to stressful circumstances [2,19].

Although structured and predictable daily activities may support the organization of family life and serve as a protective resource under conditions of increased burden, evidence on the role of family routines among families of children with intellectual disabilities remains limited. Research involving children with neurodevelopmental delays suggests that more frequent family routines are associated with lower levels of child externalizing behaviors and may buffer parental stress in some circumstances [6]. Previous research in this population has focused primarily on parenting stress, parental mental health, and family quality of life [8,10,11,12], whereas family routines have more often been considered one component of broader family functioning than a distinct area of investigation.

Furthermore, few studies have simultaneously examined the frequency of family routines, the importance parents attribute to these routines, and their associations with parenting stress, particularly through comparisons of families of children with and without intellectual disabilities. In addition, routine domains, such as weekday, leisure-time, and disciplinary routines, have received comparatively limited attention as distinct aspects of everyday family functioning. These gaps underscore the need for research examining how family routines relate to family functioning and parental well-being in families raising children with intellectual disabilities.

Accordingly, this study aimed to examine parenting stress and the frequency and perceived importance of family routines among families of children with and without intellectual disabilities, as well as the associations between family routines and parenting stress. Particular attention was given to weekday, leisure-time, and disciplinary routines in order to examine group differences and their associations with parenting stress.

## 2. Materials and Methods

### 2.1. Study Design and Setting

This study employed a cross-sectional design with comparative and correlational components. Data were collected using two self-report questionnaires administered in several cities and suburban areas in the northern region of the Republic of Serbia.

### 2.2. Participants

A convenience sampling method was employed. A total of 150 questionnaires were distributed to parents, of which 141 were returned. Sixteen returned questionnaires (11.3%) were excluded because they contained missing responses that prevented the calculation of complete FTRI and/or PSS scores. No imputation of missing questionnaire data was performed, and these cases were excluded from the analyses.

Of the total sample, 106 participants (84.8%) were mothers and 19 (15.2%) were fathers. The sample comprised 75 parents of typically developing (TD) children and 50 parents of children with intellectual disabilities (ID). Sociodemographic and family characteristics of the participants, presented separately by study group, are shown in Table 1.

The mean age of the children was 7.84 years (SD = 0.80). Among children with intellectual disabilities, 39 (78.0%) had mild intellectual disabilities and 11 (22.0%) had moderate intellectual disabilities. All children in the intellectual disability group had a formal diagnosis documented in their medical records. Permission to review the relevant medical documentation was obtained from the parents. Of the 50 children with intellectual disabilities, 47 attended special schools, whereas three attended mainstream schools.

### 2.3. Procedure

Information regarding the purpose of the study and the participation procedures was provided through written flyers distributed in schools and kindergartens, direct contact with potential participants, and collaboration with parent associations. These materials described the study objectives, the questionnaires used, instructions for completing the questionnaires, the guaranteed anonymity of participants, the voluntary nature of participation, and the right to withdraw from the study at any time without consequences.

Prior to completing the questionnaires, all participants received written and verbal information about the study and provided written informed consent.

The questionnaires were administered in paper-and-pencil format. Parents who were informed about the study through schools or kindergartens attended a scheduled meeting with a researcher, during which they completed the questionnaires. The entire procedure was conducted in a designated private room, and the completed questionnaires were returned directly to the researcher.

For parents who were members of associations of parents of children with intellectual disabilities, the researcher personally delivered the questionnaires, explained the purpose of the study and the instructions for completion, and offered participants the option of returning the completed questionnaires either by mail or in person to the researcher.

### 2.4. Instruments

Data were collected using a general sociodemographic questionnaire and two self-report instruments. The general questionnaire collected sociodemographic and family information, including parent age and gender, place of residence, marital status, number of children, educational level, employment status, satisfaction with partner support, and perceived socioeconomic status. Information on the presence and severity of intellectual disability in the child was also recorded. Family routines were assessed using the Family Time and Routines Index [20]. The instrument comprises 32 items organized into eight subscales: weekday and leisure routines (10 items; e.g., “Family goes some place special together each week”), parental routines (4 items; e.g., “Parents have time with each other quite often”), family bedtime routine (2 items; e.g., “Children go to bed at the same time almost every night”), family meals (2 items; e.g., “Whole family eats one meal together daily”), extended family routines (4 items; e.g., “Family has regular visits with relatives”), coming and going routines (4 items; e.g., “Family checks in or out with each other when someone leaves or comes home”), family disciplinary routines (2 items; e.g., “Parents have certain things they almost always do when children get out of line”), and household chores (4 items; e.g., “Children do regular household chores”). For the Family Routine Frequency Index, respondents rated the occurrence of each routine on a 4-point scale ranging from 0 (the routine is not present in the family) to 3 (the routine is consistently present or occurs regularly). Thus, higher scores indicated greater routinization and regularity of family activities. For the Family Routine Importance Index, respondents rated the perceived importance of each routine on a 4-point scale ranging from 0 (not important) to 3 (very important), with higher scores indicating greater perceived importance of the routine for family functioning. The total importance score was calculated by summing the 32 item scores and could range from 0 to 96. As no published Serbian validation of the Family Time and Routines Index (FTRI) was identified, the instrument was translated and culturally adapted for the purposes of the present study using forward translation, back-translation, and expert review. Internal consistency was assessed in the present sample; however, this procedure should not be considered a comprehensive psychometric validation of the Serbian version of the FTRI.

Parenting stress was assessed using the Parental Stress Scale (PSS) [21]. The scale consists of 18 items measuring both positive and negative aspects of the parental role, conceptualizing parenting as a source of both stress and satisfaction. The items cover several domains, including parental rewards, emotional burden, time demands, and perceived resources available for parenting. Example items include statements such as “I feel overwhelmed by the responsibilities of being a parent” (negative item) and “I am happy in my role as a parent” (positive item, reverse-scored). Participants rate each item on a 5-point Likert scale ranging from 1 (strongly disagree) to 5 (strongly agree). After reverse coding of positively worded items, higher scores indicate higher levels of parenting stress. The total score ranges from 18 to 90, with higher scores reflecting greater perceived stress in the parenting role. The total PSS score was calculated as the sum of all 18 items after reverse scoring the positively worded items, in accordance with the original scoring procedure [21]. The PSS was translated into Serbian and back-translated into English by independent bilingual translators. The translated version was reviewed for conceptual and linguistic equivalence prior to administration. As no published Serbian validation was identified, its psychometric properties were evaluated in the present sample.

### 2.5. Statistical Analysis

Data were analyzed using IBM SPSS Statistics, version 28 (IBM Corp., Armonk, NY, USA). Descriptive statistics included frequencies and percentages for categorical variables, means and standard deviations for approximately normally distributed numerical variables, and medians and interquartile ranges for ordinal or non-normally distributed variables. The internal consistency of the Family Time and Routines Index and the Parental Stress Scale was assessed in the present sample. Cronbach’s alpha was calculated for the total FTRI frequency and importance indices and for subscales comprising four or more items. For the three two-item FTRI subscales (Family Bedtime Routine, Family Meals, and Family Disciplinary Routines), the Spearman–Brown coefficient was used as an estimate of internal consistency. Normality of the distributions was assessed using the Kolmogorov–Smirnov test, skewness and kurtosis indices, and visual inspection of the distributions. Analyses were based on available data, with no imputation of missing values.

Between-group differences were examined using the Mann–Whitney U test for ordinal and non-normally distributed variables and Pearson’s chi-square test for categorical variables. When expected cell frequencies were insufficient for the chi-square approximation in contingency tables with more than two categories, the Fisher–Freeman–Halton exact test was used. Kruskal–Wallis tests were used when parenting stress was compared across nominal variables with more than two categories. Spearman rank-order correlations were used to assess associations involving ordinal or continuous variables for which a nonparametric approach was appropriate. Effect sizes for Mann–Whitney comparisons were calculated as *r* = |*Z*|/√*N*, while Cramér’s *V* was reported for categorical group comparisons.

To control the false discovery rate arising from multiple testing, the Benjamini–Hochberg procedure was applied separately within each study group across the 10 tests examining associations between parenting stress and sociodemographic and family characteristics. The Benjamini–Hochberg procedure was also applied separately to the nine between-group comparisons of family-routine frequency and, within each study group, to the eight domain-level associations between parenting stress and the perceived importance of family routines. Adjusted *p* values are reported for these families of tests. The complete results of these analyses, presented separately for the TD and ID groups, are provided in Supplementary Table S1.

A hierarchical multiple regression analysis was conducted in the pooled sample to examine the incremental contribution of family routines to parenting stress. Covariates were selected on substantive grounds to account for child, parental, household, relational, and socioeconomic characteristics plausibly associated with parenting demands, family resources, and the organization of family routines, rather than on the basis of between-group statistical significance alone. Study group, child age, parental education, employment status, household size, satisfaction with partner support, marital status, and satisfaction with socioeconomic status were entered in the first block. Parental education was dichotomized as primary/secondary education (0) versus higher/university education (1). This dichotomization was used because only one participant reported primary education, making reliable estimation of the four original educational categories impractical and avoiding the assumption of a linear relationship across the four ordered categories. Employment status was coded as unemployed (0) and employed (1). Satisfaction with partner support was assessed on a scale ranging from 0 to 5, with higher scores indicating greater satisfaction. Satisfaction with socioeconomic status was treated as a continuous variable, with higher scores indicating greater satisfaction. Total family-routine frequency and importance scores were entered in the second block.

Regression diagnostics included assessment of multicollinearity, leverage, and influential observations, as well as visual inspection of the normal P–P plot and a plot of standardized residuals against standardized predicted values. Variance inflation factors were below 4. Visual inspection of the residual plot did not indicate a clear systematic pattern suggestive of substantial nonlinearity or pronounced heteroscedasticity, and the normal P–P plot showed only minor departures from normality. Although one relatively large standardized residual was observed, no highly influential observations were identified based on Cook’s distance (maximum Cook’s distance = 0.075).

As a sensitivity analysis, the hierarchical regression was repeated using a more parsimonious adjustment set that excluded marital status and satisfaction with socioeconomic status to assess whether the principal associations with family-routine frequency and importance were sensitive to the inclusion of these additional covariates.

To examine whether the associations between family routines and parenting stress differed between the TD and ID groups, two separate adjusted interaction models were estimated in the pooled sample. Prior to computing the interaction terms, the total routine frequency and importance scores were standardized as z scores (M = 0, SD = 1). Study group was coded as a binary variable (0/1). The first model included study group, standardized routine frequency, the group × routine frequency interaction term, and the same covariates used in the fully adjusted hierarchical regression model (child age, parental education, employment status, household size, satisfaction with partner support, marital status, and satisfaction with socioeconomic status). Routine importance was not included in this model. The second model used the same covariate set and included study group, standardized routine importance, and the group × routine importance interaction term; routine frequency was not included in this model. Thus, each interaction model evaluated moderation of one routine measure separately.

### 2.6. Ethical Considerations

The study was conducted in accordance with the ethical principles for research involving human participants and the Declaration of Helsinki. Ethical approval was obtained from the Ethics Committee for Clinical Research of the Faculty of Medicine, University of Novi Sad, Serbia (Protocol Code: 01-39/75). All participants received information about the study aims, procedures, voluntary nature of participation, anonymity, and their right to withdraw without consequences. Written informed consent was obtained from all participants prior to data collection.

## 3. Results

### 3.1. Reliability of the Measures

Reliability analysis indicated high internal consistency for both FTRI total indices, with Cronbach’s α = 0.914 for the Family Routine Frequency Index and α = 0.922 for the Family Routine Importance Index. For the frequency subscales comprising four or more items, Cronbach’s alpha coefficients were 0.798 for Weekday and Leisure Routines, 0.688 for Parental Routines, 0.680 for Extended Family Routines, 0.609 for Coming and Going Routines, and 0.570 for Household Chores. For the two-item frequency subscales, Spearman–Brown coefficients were 0.639 for Family Bedtime Routine, 0.747 for Family Meals, and 0.824 for Family Disciplinary Routines. For the importance subscales comprising four or more items, Cronbach’s alpha coefficients were 0.862 for Weekday and Leisure Routines, 0.784 for Parental Routines, 0.678 for Extended Family Routines, 0.603 for Coming and Going Routines, and 0.826 for Household Chores. For the two-item importance subscales, Spearman–Brown coefficients were 0.495 for Family Bedtime Routine, 0.675 for Family Meals, and 0.596 for Family Disciplinary Routines. The Parental Stress Scale demonstrated acceptable internal consistency (Cronbach’s α = 0.76).

### 3.2. Sample Characteristics and Group Comparisons

Sociodemographic and family characteristics of the TD and ID groups are presented in Table 1. Significant between-group differences were observed in child age, number of children, household size, place of residence, marital status, parental education, and satisfaction with socioeconomic status (all *p* < 0.05). No significant differences were found in parent gender, parent age, employment status, or satisfaction with partner support (all *p* > 0.05).

### 3.3. Group Differences in Parenting Stress

Parents of children with intellectual disabilities (Mdn = 54, IQR = 10) reported significantly higher levels of parenting stress than parents of typically developing children (Mdn = 31, IQR = 11), U = 118.00, Z = −8.86, *p* < 0.001, *r* = 0.79, indicating a large effect size (Table 2).

### 3.4. Associations of Parenting Stress with Family Routines and Sociodemographic Characteristics

Among parents of typically developing children, parenting stress was negatively associated with both the frequency of family routines (Spearman’s *r*_s_ = −0.493, *p* < 0.001) and their perceived importance (*r*_s_ = −0.254, *p* = 0.028).

Analyses of associations between parenting stress and sociodemographic and family characteristics indicated nominal associations with employment status (*p* = 0.009) and place of residence (*p* = 0.021). However, neither association remained statistically significant after Benjamini–Hochberg correction was applied across the 10 sociodemographic and family-characteristic tests within the TD group (adjusted *p* = 0.090 and 0.105, respectively). No other sociodemographic or family characteristic examined was significantly associated with parenting stress after correction for multiple testing.

Among parents of children with intellectual disabilities, parenting stress was negatively associated with both the frequency of family routines (*r*_s_ = −0.493, *p* < 0.001) and their perceived importance (*r*_s_ = −0.329, *p* = 0.020).

Analyses of associations between parenting stress and sociodemographic and family characteristics indicated nominal negative associations with satisfaction with partner support (*r*_s_ = −0.311, *p* = 0.030) and satisfaction with socioeconomic status (*r*_s_ = −0.359, *p* = 0.010). However, neither association remained statistically significant after Benjamini–Hochberg correction was applied across the 10 sociodemographic and family-characteristic tests within the ID group (adjusted *p* = 0.150 and 0.100, respectively). No other sociodemographic or family characteristic examined was significantly associated with parenting stress after correction for multiple testing.

Complete group-specific results for the associations between parenting stress and the 10 sociodemographic and family characteristics, including raw and Benjamini–Hochberg-adjusted *p* values, are presented in Supplementary Table S1.

### 3.5. Group Differences in Family Time and Routines

Mann–Whitney U tests revealed significant differences between the TD and ID groups across all family routine dimensions and the total routine frequency score (Table 3).

Routine frequency was significantly higher in the TD group across all eight family routine dimensions and for the total routine frequency score. All nine group differences remained statistically significant after Benjamini–Hochberg correction (FDR-adjusted *p* ≤ 0.002), with effect sizes ranging from *r* = 0.28 to *r* = 0.86.

Regarding the perceived importance of family routines, the median total importance score was 55.00 (IQR = 8.00) in the TD group and 37.50 (IQR = 12.25) in the ID group.

### 3.6. Associations Between Parenting Stress and the Perceived Importance of Family Routines by Study Group

Spearman rank-order correlations were conducted separately within the TD and ID groups to examine associations between parenting stress and the perceived importance of individual family routine dimensions (Table 4). In the TD group, none of the associations were statistically significant. In the ID group, parenting stress was negatively associated with the perceived importance of weekday and leisure routines (*r*_s_ = −0.377, *p* = 0.007) and family disciplinary routines (*r*_s_ = −0.446, *p* = 0.001). Both associations remained statistically significant after Benjamini–Hochberg correction was applied across the eight domain-level comparisons within the ID group (adjusted *p* = 0.028 and 0.008, respectively).

### 3.7. Parental Stress Prediction

A hierarchical multiple regression analysis was conducted in the pooled sample (*N* = 125) to examine whether family-routine frequency and perceived importance explained additional variance in parenting stress beyond study group and the selected covariates. Study group, child age, parental education, employment status, household size, satisfaction with partner support, marital status, and satisfaction with socioeconomic status were entered in the first step. The first-step model explained 67.8% of the variance in parenting stress (R^2^ = 0.678, adjusted R^2^ = 0.655, F(8, 116) = 30.47, *p* < 0.001). Total family-routine frequency and perceived importance were entered in the second step. Their addition explained a further 6.6% of the variance in parenting stress (ΔR^2^ = 0.066, ΔF(2, 114) = 14.80, *p* < 0.001). The final model explained 74.4% of the variance in parenting stress (R^2^ = 0.744, adjusted R^2^ = 0.722, F(10, 114) = 33.13, *p* < 0.001).

In the final model, greater family-routine frequency was independently associated with lower parenting stress (B = −0.386, SE = 0.081, β = −0.415, 95% CI [−0.547, −0.224], *p* < 0.001), whereas perceived importance of family routines was not independently associated with parenting stress (B = 0.027, SE = 0.114, β = 0.023, 95% CI [−0.198, 0.253], *p* = 0.810). Study group also remained independently associated with parenting stress (B = −15.028, SE = 2.235, β = −0.548, 95% CI [−19.455, −10.601], *p* < 0.001). None of the remaining covariates were statistically significant in the final model (Table 5).

Regression diagnostics did not indicate substantial violations of model assumptions. Visual inspection of standardized residuals plotted against standardized predicted values showed no clear evidence of substantial nonlinearity or pronounced heteroscedasticity, while the normal P–P plot showed only minor departures from normality. Although one relatively large standardized residual was observed, Cook’s distance values did not indicate highly influential observations (maximum Cook’s distance = 0.075). Multicollinearity was not considered problematic, with all variance inflation factors below 4.

The findings were robust to a more parsimonious covariate specification. When marital status and satisfaction with socioeconomic status were excluded from the adjustment set, greater family-routine frequency remained independently associated with lower parenting stress (B = −0.372, SE = 0.075, β = −0.400, 95% CI [−0.521, −0.222], *p* < 0.001), whereas routine importance remained nonsignificant (B = 0.014, SE = 0.111, β = 0.011, 95% CI [−0.207, 0.234], *p* = 0.902). Thus, the principal findings were not materially altered by the alternative covariate specification.

Two separate adjusted interaction models were examined to determine whether the associations of family-routine frequency and perceived importance with parenting stress differed between the TD and ID groups. Both models included the full covariate set and were based on the pooled sample (*N* = 125). The group × routine frequency interaction was not statistically significant (B = −0.026, SE = 1.993, β = −0.001, 95% CI [−3.974, 3.922], *p* = 0.990). Similarly, the group × routine importance interaction was not statistically significant (B = −0.649, SE = 2.290, β = −0.026, 95% CI [−5.185, 3.887], *p* = 0.777). Thus, the interaction analyses provided no evidence that the associations between either routine frequency or routine importance and parenting stress differed between the TD and ID groups.

## 4. Discussion

The present study examined parenting stress, the presence and perceived importance of family routines in families of children with and without intellectual disabilities, and the associations between family routines and parenting stress. Particular attention was given to different domains of family routines, including weekday routines, leisure-time routines, and discipline-related routines.

The findings demonstrated that parents of children with intellectual disabilities reported significantly higher levels of parenting stress compared with parents of typically developing children. In addition, families of children with intellectual disabilities reported lower frequency of family routines across the assessed domains. Importantly, higher frequency of family routines was associated with lower levels of parenting stress in both groups, and family routine frequency remained a significant predictor of parenting stress after controlling for sociodemographic characteristics. These findings suggest that the organization and regularity of everyday family activities may represent an important resource associated with parental well-being, particularly in families experiencing increased caregiving demands [2,6,9,10].

Consistent with previous research, the elevated levels of parenting stress observed among parents of children with intellectual disabilities may be understood in relation to the complex and ongoing demands associated with caregiving. These demands may be particularly pronounced when children have substantial support needs or co-occurring health and behavioural difficulties. They can include providing intensive support with daily living activities, coordinating medical, educational, and rehabilitation services, and adapting family activities to the child’s individual developmental needs [10,11,22,23].

The experience of parenting stress in families of children with intellectual disabilities is influenced not only by child characteristics but also by the broader family and contextual circumstances in which caregiving occurs. Greater child functional impairment, which may require more intensive parental support, has been associated with an increased likelihood of clinically significant parenting stress among parents of children with neurodevelopmental disorders [24]. In families of children with intellectual disabilities, the need for continuing care and collaboration with health, educational, and rehabilitation professionals highlights the importance of family-centred approaches that recognize family needs, support family functioning, and involve parents in care planning [11,23].

From a family systems perspective, these findings underscore the importance of considering not only the challenges experienced by families of children with intellectual disabilities but also the family processes that may support adaptation. Family routines can be understood as an important component of everyday family organization, potentially providing a more predictable and structured context for managing increased caregiving demands.

The association between family routines and parenting stress observed in this study is consistent with previous literature indicating that predictable and structured patterns of everyday family activities may be associated with family adaptation in the context of increased stress and caregiving demands. Family routines may serve as an organizing framework for daily life, supporting the management of ongoing demands, emotional support exchanges, and a sense of normalcy when families face chronic challenges. In this way, the presence and regularity of routines may be related to parents’ perceptions of structure, competence, and control in managing everyday caregiving demands [7,15].

In the present study, family routine frequency was negatively associated with parenting stress in both groups, such that parents who reported more frequent routines also reported lower levels of parenting stress. Furthermore, in the pooled hierarchical regression analysis, greater family routine frequency remained independently associated with lower parenting stress after accounting for study group and selected sociodemographic and family characteristics. These findings suggest that the organization and regularity of everyday family life may be relevant to parental well-being. However, given the cross-sectional design, no causal inferences can be drawn. The association may be reciprocal: more consistent routines may be linked to better stress regulation, whereas lower parenting stress may enable parents to establish and maintain routines more effectively.

The role of family routines can also be understood within Walsh’s family resilience framework, which emphasizes families’ capacity to mobilize resources and adapt to adversity. Research involving parents of children with intellectual disabilities further indicates that stress and adaptation are shaped by caregiving demands, family resources, and parental well-being [1,25,26]. Within this perspective, routines may be conceptualized as one potential organizational resource through which families may manage increased demands and support adaptation [2,7,27]. Similarly, the Family Adjustment and Adaptation Response (FAAR) model proposes that family adaptation reflects the balance between family demands and the capabilities available to manage them, including resources and coping processes. This perspective is particularly relevant to families of children with intellectual disabilities, for whom parenting stress and family quality of life have been associated with caregiving demands, family resources, and broader family functioning [10,19].

The present findings further suggest that different dimensions of family routines may have distinct relevance for parental stress. At the domain level, no associations between parenting stress and perceived importance remained statistically significant in the TD group after correction for multiple testing. In the ID group, parenting stress was negatively associated with the perceived importance of weekday and leisure routines and family disciplinary routines, and both associations remained significant after Benjamini–Hochberg correction. When routine frequency and perceived importance were examined together in the pooled regression model, routine frequency was independently associated with parenting stress, whereas perceived importance did not make a significant independent contribution. This finding suggests that the actual implementation and regularity of routines may have particular relevance to parents’ experiences of stress, as routine frequency remained independently associated with parenting stress when perceived importance was considered simultaneously.

The present findings suggest that weekday and leisure-time routines may be relevant domains of family organization in relation to parenting stress. Weekday routines, including activities related to meals, sleep, hygiene, and everyday communication, may provide structure and predictability in family life. For families experiencing increased caregiving demands, predictable daily patterns may facilitate the coordination of responsibilities and reduce the cognitive demands associated with repeated everyday decision-making. Previous research has similarly suggested that consistent routines may support family functioning by increasing predictability and reducing uncertainty in everyday interactions [7,28].

Weekday and leisure-time routines may provide a context for shared family activities and interaction. Regular patterns of spending free time together may be associated with opportunities for connection and positive family experiences beyond everyday caregiving tasks. In families of children with intellectual disabilities, participation in leisure activities may be shaped by children’s developmental characteristics and support needs, as well as by the resources and opportunities available to the family. Thus, the development and maintenance of feasible leisure-time routines may be relevant to the broader organization of family life under increased caregiving demands [7,15].

Discipline-related routines require particular consideration, as group differences should be interpreted in light of children’s developmental characteristics and the specific demands experienced by their families. In the present study, discipline-related routines were reported more frequently by parents of typically developing children. This difference may reflect variations in children’s developmental and adaptive functioning, communication abilities, behavioural characteristics, and capacity to understand and respond to behavioural expectations, although these factors were not directly assessed in the present study. Previous research suggests that parenting practices and parent–child interactions in families of children with intellectual disabilities may be shaped by children’s behavioural characteristics and support needs [29,30,31]. Thus, the lower reported frequency of discipline-related routines should not be interpreted as evidence of less parental structure or less effective parenting; rather, it may reflect developmentally responsive adaptations in how behavioural expectations and guidance are organised within the family context.

Taken together, the findings of this study underscore the relevance of family routines as an important aspect of family functioning and a potential focus of family-centred support. Because more frequent family routines were associated with lower parenting stress and remained independently associated with parenting stress after adjustment for selected sociodemographic characteristics, supporting everyday family organisation may be a relevant component of family-centred services for families experiencing increased caregiving demands. However, longitudinal and intervention-based research is needed to determine whether supporting the development and maintenance of family routines leads to reductions in parenting stress over time.

For families of children with intellectual disabilities, routine-based support may focus on identifying existing family strengths, facilitating the development of realistic and sustainable daily patterns, and adapting routines to the child’s developmental abilities, communication needs, and individual characteristics [32,33]. Interventions are more likely to be feasible and meaningful when they are developed from an understanding of families’ everyday activities, priorities, values, and contextual constraints [33]. Rather than promoting rigid structures, such approaches should emphasise flexibility and responsiveness, enabling families to maintain predictability while adapting to changing circumstances, caregiving demands, and developmental transitions [34,35].

Family-centred support should also consider the broader context in which routines are established and maintained. Socioeconomic circumstances, the availability of informal and formal social support, access to health, rehabilitation, and educational services, and parents’ coping resources may influence families’ capacity to organise everyday activities and manage caregiving demands. Accordingly, individualised approaches that address both family routines and parents’ experiences of stress may be relevant to supporting family adaptation and resilience [10,13,18,23].

Overall, this study contributes to understanding the associations between family routines and parenting stress among families of children with and without intellectual disabilities. The findings suggest that the organisation of everyday routines may be an important dimension of family functioning that warrants further attention in research and clinical practice. Longitudinal studies are needed to clarify the temporal direction of the associations between family routines and parenting stress. In addition, intervention studies should examine whether supporting the development and maintenance of specific routine domains is associated with improvements in parental well-being and broader family outcomes.

### Limitations and Recommendations for Future Research

This study has several limitations that should be considered when interpreting the findings. First, the sample size was relatively small, and the two study groups were unequal in size. In addition, the group of children with intellectual disabilities predominantly comprised children with mild intellectual disabilities. Future research should recruit larger and more balanced samples, include children with a broader range of intellectual disability severity, and examine whether associations between family routines and parenting stress differ across developmental and support profiles.

Second, the use of a convenience sample recruited from a limited geographical area may limit the generalisability of the findings. The sample also consisted predominantly of mothers (84.8%), limiting the extent to which the findings can be generalised to fathers and precluding a reliable examination of parent-gender differences. Future studies should recruit more geographically diverse samples and include sufficient numbers of mothers and fathers to examine potential differences in family routines and parenting stress.

Third, although the total FTRI frequency and importance indices demonstrated high internal consistency, reliability was lower for several individual subscales. In particular, lower coefficients were observed for the frequency scores of Household Chores and Coming and Going Routines, as well as for the importance scores of Family Bedtime Routine, Family Disciplinary Routines, and Coming and Going Routines. The Family Bedtime and Family Disciplinary subscales comprise only two items, for which reliability estimates should be interpreted with particular caution. Accordingly, findings involving these individual routine domains should be interpreted cautiously. In addition, both family routines and parenting stress were assessed using parent-report measures, which may be affected by shared-method variance, recall bias, and parents’ current emotional states. Future research would benefit from combining parent-report questionnaires with observational assessments of family interactions and routines, reports from multiple caregivers, and, where appropriate, information from teachers or service providers. Although the regression models adjusted for several relevant child, parental, relational, household, and socioeconomic characteristics, residual confounding by unmeasured or imperfectly measured factors cannot be excluded.

Finally, the cross-sectional design precludes conclusions regarding the temporal direction or causality of the associations between family routines and parenting stress. Longitudinal research is needed to examine how family routines and parenting stress change over time and whether changes in routine frequency prospectively predict changes in parenting stress. Intervention studies should also investigate whether family-centred support for developing and maintaining feasible routines is associated with reductions in parenting stress and improvements in family functioning.

## 5. Conclusions

This study contributes to understanding the associations between family routines and parenting stress among families of children with and without intellectual disabilities. Parents of children with intellectual disabilities reported higher levels of parenting stress than parents of typically developing children, while families of typically developing children reported more frequent family routines across all assessed domains.

Higher family routine frequency was associated with lower parenting stress in both groups and remained independently associated with parenting stress in the pooled regression model after accounting for study group and selected sociodemographic and family characteristics. In contrast, the perceived importance of family routines did not make a significant independent contribution when routine frequency was considered. These findings indicate that the organization and regularity of everyday family activities may be an important aspect of family functioning associated with parental well-being.

However, because this was a cross-sectional study, the direction and causality of the association between family routine frequency and parenting stress cannot be determined. Routine-based, family-centred support may warrant further investigation as a potential component of services for families of children with intellectual disabilities, but this study did not establish that such support reduces parenting stress. Longitudinal and intervention studies are needed to determine whether supporting the development and maintenance of feasible, adaptive family routines contributes to reductions in parenting stress and improvements in family functioning.

## Figures and Tables

**Table 1:** Sociodemographic and family characteristics of participants according to study group.

Characteristic	TD (*n* = 75)	ID (*n* = 50)	*p*	Effect Size
Parent gender, *n* (%)			0.476 ^a^	0.06
Father	10 (13.3)	9 (18.0)		
Mother	65 (86.7)	41 (82.0)		
Parent age, *n* (%)			0.342 ^b^	0.17
20–30 years	4 (5.3)	0 (0.0)		
30–40 years	27 (36.0)	21 (42.0)		
41–50 years	38 (50.7)	23 (46.0)		
>51 years	6 (8.0)	6 (12.0)		
Child age, M ± SD	8.08 ± 0.82	7.48 ± 0.61	<0.001 ^c^	0.36
Number of children, Mdn (IQR)	2 (1)	1 (1)	0.007 ^c^	0.24
Household size, Mdn (IQR)	3 (1)	3 (1)	<0.001 ^c^	0.33
Place of residence, *n* (%)			0.010 ^a^	0.23
Urban	35 (46.7)	35 (70.0)		
Rural	40 (53.3)	15 (30.0)		
Marital status, *n* (%)			<0.001 ^b^	0.35
Married	67 (89.3)	34 (68.0)		
Divorced	1 (1.3)	11 (22.0)		
Cohabiting	7 (9.3)	5 (10.0)		
Parental education, *n* (%)			0.003 ^b^	0.32
Primary	0 (0.0)	1 (2.0)		
Secondary	11 (14.7)	18 (36.0)		
Higher	40 (53.3)	25 (50.0)		
University	24 (32.0)	6 (12.0)		
Employment status, *n* (%)			0.068 ^a^	0.16
Employed	64 (85.3)	36 (72.0)		
Unemployed	11 (14.7)	14 (28.0)		
Satisfaction with partner support, Mdn (IQR)	4 (1)	4 (2) ^d^	0.129 ^c^	0.14
Satisfaction with socioeconomic status, Mdn (IQR)	4 (1)	3 (1)	0.023 ^c^	0.20

Note: TD = parents of typically developing children; ID = parents of children with intellectual disabilities; M = mean; SD = standard deviation; Mdn = median; IQR = interquartile range. ^a^ Pearson’s chi-square test. ^b^ Fisher–Freeman–Halton exact test. ^c^ Mann–Whitney U test. ^d^ *n* = 49 due to one missing value. Effect sizes are reported as Cramér’s V for categorical comparisons and r = |Z|/√N for Mann–Whitney U tests.

**Table 2:** Group differences in parenting stress between parents of typically developing children and parents of children with intellectual disabilities.

Variable	TD Mdn (IQR)	ID Mdn (IQR)	U	Z	*p*	Effect Size
Parenting stress	31 (11)	54 (10)	118.00	−8.86	<0.001	0.79

Note: TD = parents of typically developing children; ID = parents of children with intellectual disabilities. Effect size (*r*) was calculated as *r* = |*Z*|/√*N*.

**Table 3:** Group differences in family routine frequency between parents of typically developing children and parents of children with intellectual disabilities.

Family Routine Dimension	TD Mdn (IQR)	ID Mdn (IQR)	*U*	*Z*	*p*	FDR *p*	Effect Size (*r*)
Weekday and leisure routines	22.00 (6.00)	15.00 (5.25)	619.50	−6.34	<0.001	<0.001	0.57
Parental routines	6.00 (4.00)	4.00 (3.00)	725.00	−5.84	<0.001	<0.001	0.52
Family bedtime routine	5.00 (2.00)	4.00 (2.00)	1014.00	−4.45	<0.001	<0.001	0.40
Family meals	5.00 (1.00)	4.00 (1.00)	1272.00	−3.11	0.002	0.002	0.28
Extended family routines	9.00 (4.00)	7.00 (2.00)	1120.50	−3.84	<0.001	<0.001	0.34
Coming and going routines	10.00 (3.00)	7.50 (4.00)	884.00	−5.05	<0.001	<0.001	0.45
Family disciplinary routines	6.00 (1.00)	2.00 (2.00)	21.50	−9.61	<0.001	<0.001	0.86
Household chores	9.00 (3.00)	6.00 (2.00)	551.00	−6.72	<0.001	<0.001	0.60
Total routines	70.00 (15.00)	47.50 (15.00)	369.50	−7.59	<0.001	<0.001	0.68

Note: TD = parents of typically developing children; ID = parents of children with intellectual disabilities; Mdn = median; IQR = interquartile range; FDR = false discovery rate. Effect size (*r*) was calculated as *r* = |*Z*|/√*N*. FDR-adjusted *p* values were obtained using the Benjamini–Hochberg procedure.

**Table 4:** Spearman rank-order correlations between parenting stress and the perceived importance of family routine dimensions by study group.

Family Routine Dimension	TD *r*_s_	TD *p*	TD FDR *p*	ID *r*_s_	ID *p*	ID FDR *p*
Weekday and leisure routines	−0.153	0.189	0.302	−0.377	0.007	0.028
Parental routines	−0.126	0.280	0.373	−0.237	0.098	0.261
Family bedtime routine	−0.189	0.104	0.302	−0.075	0.604	0.795
Family meals	−0.156	0.181	0.302	−0.071	0.625	0.795
Extended family routines	−0.096	0.415	0.474	−0.085	0.555	0.795
Coming and going routines	−0.212	0.068	0.302	0.035	0.811	0.811
Family disciplinary routines	−0.069	0.558	0.558	−0.446	0.001	0.008
Household chores	−0.157	0.179	0.302	−0.057	0.696	0.795

Note: TD = parents of typically developing children; ID = parents of children with intellectual disabilities; FDR = false discovery rate. FDR-adjusted *p* values were calculated separately within each study group using the Benjamini–Hochberg procedure across the eight family routine dimensions.

**Table 5:** Hierarchical multiple regression analysis of parenting stress in the pooled sample.

Predictor	B	SE	β	95% CI for B	*p*
Step 1					
Study group	−20.678	1.697	−0.754	[−24.039, −17.318]	<0.001
Parental education	−0.406	1.840	−0.013	[−4.050, 3.238]	0.826
Employment status	2.650	1.908	0.079	[−1.130, 6.430]	0.168
Marital status	−2.822	1.974	−0.083	[−6.731, 1.087]	0.155
Child age	0.127	1.054	0.007	[−1.961, 2.215]	0.904
Household size	−0.039	0.737	−0.003	[−1.499, 1.420]	0.958
Satisfaction with partner support	−0.950	0.717	−0.077	[−2.371, 0.470]	0.188
Satisfaction with socioeconomic status	−0.660	0.942	−0.041	[−2.526, 1.205]	0.485
Step 2					
Study group	−15.028	2.235	−0.548	[−19.455, −10.601]	<0.001
Parental education	−0.984	1.665	−0.031	[−4.282, 2.313]	0.555
Employment status	1.949	1.725	0.058	[−1.468, 5.367]	0.261
Marital status	−0.238	1.838	−0.007	[−3.879, 3.403]	0.897
Child age	−0.969	0.969	−0.050	[−2.888, 0.949]	0.319
Household size	−0.580	0.671	−0.047	[−1.910, 0.749]	0.389
Satisfaction with partner support	−0.169	0.670	−0.014	[−1.497, 1.158]	0.801
Satisfaction with socioeconomic status	0.641	0.892	0.039	[−1.125, 2.407]	0.474
Total family-routine frequency	−0.386	0.081	−0.415	[−0.547, −0.224]	<0.001
Total family-routine importance	0.027	0.114	0.023	[−0.198, 0.253]	0.810

Note: Model statistics. Step 1: R^2^ = 0.678, adjusted R^2^ = 0.655, F(8, 116) = 30.47, *p* < 0.001. Step 2: R^2^ = 0.744, adjusted R^2^ = 0.722, ΔR^2^ = 0.066, ΔF(2, 114) = 14.80, *p* < 0.001. B = unstandardized regression coefficient; SE = standard error; β = standardized regression coefficient; CI = confidence interval. Parental education was coded 0 = primary/secondary and 1 = higher/university; employment status was coded 0 = unemployed and 1 = employed. Higher scores for satisfaction with partner support and socioeconomic status indicate greater satisfaction.

## Data Availability

The data presented in this study are not publicly available due to ethical and privacy restrictions. Anonymized data may be available from the corresponding author on reasonable request and subject to approval by the relevant ethics committee.

## Supplementary Materials

The following supporting information can be downloaded at: https://www.mdpi.com/article/10.3390/healthcare14193153/s1, Table S1: Associations between parenting stress and sociodemographic and family characteristics in the TD and ID groups.
