# Supplementary material for: Family Routines and Parenting Stress in Families of Children With and Without Intellectual Disabilities: A Cross-Sectional Study
Source: Healthcare (Basel). 2026 Sep 23;14(19):3153. doi: 10.3390/healthcare14193153 (PMC13665347; doi:10.3390/healthcare14193153)
Supplement: Supplementary file 1 [file healthcare-14-03153-s001.zip › healthcare-4493000-supplementary.pdf]

Supplementary materials

Table S1

Associations between parenting stress and sociodemographic and family characteristics in the TD and ID groups

**Characteristic**	**Test**	**TD statistic**	**TD raw p**	**TD BH-adjusted p**	**ID statistic**	**ID raw p**	**ID BH-adjusted p**
Parent gender	Mann–Whitney U	U = 324.50, Z = −0.008	.994	.994	U = 174.00, Z = −0.266	.790	.829
Parent age	Spearman	$r_s = -.136$	.244	.488	$r_s = .125$	.388	.647
Child age	Spearman	$r_s = .102$	.385	.642	$r_s = -.202$	.160	.320
Number of children	Spearman	$r_s = -.047$	.690	.863	$r_s = .073$	.616	.770
Place of residence	Mann–Whitney U	U = 482.50, Z = −2.313	**.021**	.105	U = 232.50, Z = −0.637	.524	.749
Marital status	Kruskal–Wallis	H(2) = 3.058	.217	.488	H(2) = 4.318	.115	.288
Parental education	Kruskal–Wallis	H(2) = 1.090	.580	.829	H(3) = 7.308	.063	.210
Employment status	Mann–Whitney U	U = 527.00, Z = 2.625	**.009**	.090	U = 242.00, Z = −0.217	.829	.829
Satisfaction with partner support	Spearman	$r_s = -.192$	.099	.330	$r_s = -.339$	**.016**	.080
Satisfaction with socioeconomic status	Spearman	$r_s = -.001$	.994	.994	$r_s = -.359$	**.010**	.080

Note. TD = parents of typically developing children; ID = parents of children with intellectual disability; *U* = Mann–Whitney U statistic; *Z* = standardized Mann–Whitney test statistic; *H* = Kruskal–Wallis test statistic; r_s = Spearman's rank-order correlation coefficient. BH-adjusted *p* values were calculated using the Benjamini–Hochberg procedure separately across the 10 sociodemographic and family-characteristic tests within each study group.
